# Snail1 induces epithelial-to-mesenchymal transition and tumor initiating stem cell characteristics

**DOI:** 10.1186/1471-2407-11-396

**Published:** 2011-09-19

**Authors:** Hien Dang, Wei Ding, Dow Emerson, C Bart Rountree

**Affiliations:** 1 Department of Pediatrics and Pharmacology, The Pennsylvania State University, College of Medicine, Penn State Children's Hospital, Hershey, Pennsylvania, USA

## Abstract

**Background:**

Tumor initiating stem-like cells (TISCs) are a subset of neoplastic cells that possess distinct survival mechanisms and self-renewal characteristics crucial for tumor maintenance and propagation. The induction of epithelial-mesenchymal-transition (EMT) by TGFβ has been recently linked to the acquisition of TISC characteristics in breast cancer. In HCC, a TISC and EMT phenotype correlates with a worse prognosis. In this work, our aim is to elucidate the underlying mechanism by which cells acquire tumor initiating characteristics after EMT.

**Methods:**

Gene and protein expression assays and Nanog-promoter luciferase reporter were utilized in epithelial and mesenchymal phenotype liver cancer cell lines. EMT was analyzed with migration/invasion assays. TISC characteristics were analyzed with tumor-sphere self-renewal and chemotherapy resistance assays. *In vivo *tumor assay was performed to investigate the role of Snail1 in tumor initiation.

**Conclusion:**

TGFβ induced EMT in epithelial cells through the up-regulation of Snail1 in Smad-dependent signaling. Mesenchymal liver cancer post-EMT demonstrates TISC characteristics such as tumor-sphere formation but are not resistant to cytotoxic therapy. The inhibition of *Snail1 *in mesenchymal cells results in decreased *Nanog *promoter luciferase activity and loss of self-renewal characteristics *in vitro*. These changes confirm the direct role of Snail1 in some TISC traits. *In vivo*, the down-regulation of *Snail1 *reduced tumor growth but was not sufficient to eliminate tumor initiation. In summary, TGFβ induces EMT and TISC characteristics through Snail1 and Nanog up-regulation. In mesenchymal cells post-EMT, Snail1 directly regulates *Nanog *expression, and loss of Snail1 regulates tumor growth without affecting tumor initiation.

## Background

Tumor initiating stem-like cells (TISCs), also defined as cancer stem cells, are a subpopulation of neoplastic cells that possess distinct survival and regeneration mechanisms important for chemotherapy resistance and disease progression [1,2]. By definition, TISCs possess stem cell features including resistance to apoptosis and self-renewal [3-5]. After their initial discovery and characterization within hematological malignancies [6,7], TISCs have now been described in many different malignancies including hepatocellular carcinoma (HCC) [8,9]. Further evidence supports that HCC arises as a direct consequence of dysregulated proliferation of hepatic progenitor cells [10,11]. Transcriptome analysis of HCC demonstrated that a progenitor-based (TISC-phenotype) expression profile is associated with a poor prognosis compared to differentiated tumors (hepatocyte-phenotype) [12-14].

Resistance to therapy and metastatic disease are two factors that correlate a TISC-phenotype HCC with poor survival. TISCs are hypothesized to be the source of metastatic lesions, as a tumor-initiating cell [15]. Although this hypothesis remains controversial, recent work establishes a connection between epithelial-mesenchymal-transition (EMT) and a TISC-phenotype [16,17]. EMT is a critical developmental process that plays a central role in the formation and differentiation of multiple tissues and organs. During EMT, epithelial cells lose cell-cell adhesion and apical-polarity, and acquire mesenchymal features, such as motility, invasiveness, and resistance to apoptosis [18].

One of the key hallmarks of EMT is loss of E-cadherin, a cell-adhesion protein that is regulated by multiple transcription factors including Snail, Slug, and Twist. These transcription factors act as E-box repressors and block E-cadherin transcription [18]. In cancer biology, EMT is one mechanism to explain the invasive and migratory capabilities that epithelial carcinomas acquire during metastasis [19,20]. In HCC, increased expression of the E-cadherin repressors Twist and Snail correlates with poor clinical outcomes [21]. In breast cancer, EMT is associated with the acquisition of a TISC CD44^+^/CD24^low ^phenotype [17,22].

One of the major inducer of EMT is transforming growth factor-β (TGFβ), a multifunctional cytokine that regulates cell proliferation, differentiation and apoptosis [23]. In early stages of carcinogenesis, TGFβ serves as a tumor suppressor by inhibiting cell growth, and in later stages of disease, tumor cells escape this growth inhibition. As late stage cancer tends to be resistant to TGFβ-driven growth arrest signals and as TGFβ is a known inducer of EMT, TGFβ is proposed to be a facilitator of cancer progression during late stage disease [24-26]. TGFβ induces EMT by up-regulating Snail1 via the Smad-dependent pathways [27]. Mishra and colleagues have reviewed the complexity of TGFβ signaling during hepatocarcinogenesis, specifically as related to β2-Spectrin loss and stem cell malignant transformation [15,28-30].

As additional evidence linking EMT to TISCs, TGFβ regulates *Nanog *expression, a transcription factor that contributes to self-renewal and cell fate determination in embryonic stem cells [31,32]. In prostate cancer, increased *Nanog *expression is implicated in tumor progression, and the co-expression of Nanog and Oct4 promotes tumor-sphere formation [4,33,34]. In colon cancer, increased Snail1 expression correlates to increased *Nanog *expression [35]. In human HCC cell lines, TGFβ regulates *CD133 *expression, a marker of TISCs, through induction of epigenetic modifications of the *CD133 *promoter [23,36].

Thus, several studies have demonstrated that TGFβ drives EMT through Snail1 up-regulation, and other studies have correlated EMT to the acquisition of TISC characteristics. What is lacking is an understanding of the mechanism of how liver cancer cells acquire TISC characteristics through EMT. Our hypothesis is that mesenchymal cells acquire TISC traits after EMT through Snail1-dependent mechanisms. In this report, we demonstrate that mesenchymal liver cancer cells (post-EMT) possess several TISC characteristics compared to epithelial cells. TGFβ induces EMT and TISC characteristics in epithelial cells through *Snail1*. In mesenchymal cells, knock-down of *Snail1 *results in loss of Nanog and reduction of TISC traits. *In vivo *studies demonstrate that Snail1 regulates tumor growth but does not fully control tumor initiation.

## Methods

### Cell Culture

Epithelial and mesenchymal murine liver cancer cells were cultured in Dulbecco's modified Eagle's medium (DMEM)/F12 (Sigma) supplemented with 10% fetal bovine serum as described [37]. The human HCC cell line Huh7 was provided by Jianming Huh, Penn State College of Medicine and cultured as described [36,38]. The human HCC The human HCC cell lines MHCC97-L were provided by Xinwei Wang, National Cancer Institute, under agreement with the Liver Cancer Institute, Zhongshan Hospital, Fudan University, Shanghai, China and cultured as described [39].

### Transfections

For *Snail1 *transient knockdown, cells were transfected with 100 pM of *Snail1 *Stealth siRNA (Invitrogen) using Lipofectamine 2000 (Invitrogen). For Smad signaling inhibition, cells were transfected with 2 ug of DNA using Fugene 6 (Roche). To generate *Snail1 *knockdown stable transfectants, mesenchymal cells were transfected with *Snail1 *Mission shRNA lentivirus (Sigma) and selected with 2 ug/ml of puromycin.

### Luciferase Assay

pCMV5-Smad7-HA (Plasmid 11733), pRK-Smad3ΔC (Plasmid 12626), and *Nanog-Luc *(Plasmid 16337) were provided by Addgene. Cells were plated in 12 well plates, incubated overnight, and transfected with the *Nanog-Luc *plasmid and Renilla for 24 hours (4:1 *Nanog-Luc*:Renilla ratio). Cells were washed with 1 × PBS, serum free starved for 2 hours, and treated with 5 ng/ml of TGFβ for 24 hours. Following cell lysis, luciferase activity was measured using the Dual Luciferase Assay Kit (Promega) and a Sirius Luminometer V3.1 (Zylux). Luciferase reading light units (RLU) were normalized to Renilla RLU and a fold change was calculated.

### qRT-PCR

Trizol (Invitrogen) was used to isolate total RNA from cells according to manufacturer's protocol. Isolated RNA was quantified using the ND-1000 spectrophotometer (NanoDrop) and complementary single strand DNA was synthesized using the Omniscript RT Kit according to the manufacturers protocol (Qiagen). qPCR was performed using Taqman Gene Expression Assays and ABI-Prism 7700 Thermal Cycler (Applied Biosystems). Normalization was performed using *β-actin *or *Gapdh *as an endogenous control and relative gene expression was calculated using the comparative 2^(-ΔΔCt) ^method with SDS 2.2.2 software [36].

### Cell Viability Assays

Cell viability was performed using the XTT (2,3-bis(2-methoxy-4-nitro-5-sulfophenyl)-2H-tetrazolium-5-carboxanilide) kit (Trevigen) according to the manufacturer's protocol. 5 × 10^3 ^cells were plated in 96-well plates, incubated for 24 hours at 37°C, and treated with specified agents at defined time points.

### Western Blot Analysis

Cells were washed twice with ice cold 1XPBS and cell lysates were harvested by the addition of lysis buffer (40 nM Tris [pH 7.4], 150 mM NaCl, 10 mM ethylene diamine tetraccetic acid, 10% glycerol, 1% Triton X-100, 10 mM glycerophosphate, 1 mM Na3VO4, 1 mM phenylmethylsulfonyl fluoride) supplemented with protease inhibitor cocktail tablets (Roche). BCA protein assay (Thermo Fisher Scientific) was used to determine protein concentration as described [40]. 30 ug of protein lysates were separated on a NuPAGE 4-12% Bis-Tris Gel (Invitrogen) and the separated proteins were transferred onto a polyvinylidene difluoride membrane (Invitrogen). After blocking for 60 min with 5% non-fat dry milk, membranes were incubated with the primary antibody overnight at 4°C followed by incubation with corresponding secondary antibody for 60 min at room temperature. The membranes were developed using enhance chemiluminescence solutions (Thermo Fisher Scientific) [41].

### Cell Migration Assay

The capability of tumor cell migration was assessed using a wound-healing assay. Confluent cell monolayers were manually wounded by scraping the cells with a 1,000 μL pipette tip down the center of the well. The cell culture medium was replaced and migration was assessed at 24 hours [37].

### Matrigel Invasion Assay

Cell invasion was assessed using 6-well Transwell permeable inserts with 8-μm pores (Corning) [37]. In brief, 1 × 10^5 ^cells were cultured in a serum-free DMEM/F12 medium in an insert coated with Matrigel (BD). Below the insert, the chamber of 6-well plates contained DMEM/F12 supplemented with 10% FBS. Cells were incubated in a 37°C incubator for 48 hours and the number of cells that invaded across the membranes and fallen onto the bottom of the plate was counted.

### Transcriptome analysis

Using the cell lines from the liver specific *Pten^-/- ^*model described [37] P2E (epithelial) and P2M (mesenchymal) messenger RNA were analyzed using an Illumina mouse gene chip according to the manufacturer's protocol and as described [37]. Housekeeping genes were used as standards to generate expression levels, and data analysis was conducted using 1.4-fold or greater change in expression with p < 0.05 as significant. The full complement of the expression data is available at http://www.ncbi.nlm.nih.gov/geo (Accession number GSE18255).

### Spheroid Formation Assay

The capability of self-renewal was assessed using Corning Ultra-Low Attachment Surface (Corning). 5 × 10^3 ^cells were seeded and incubated in a cell culture incubator for 1 week in DMEM/F12 supplemented with 10% FBS or serum free medium and phase-contrast images were obtained.

### In vivo tumor growth assay

Cells were counted with trypan blue exclusion and suspended in a 1:3 dilution of Matrigel (Matrigel:DMEM/F12 supplemented with 10% FBS) [36]. 1 × 10^4 ^and 1 × 10^5 ^cells/50 μL were injected subcutaneously into 10-week-old nude mice. Caliper measurements of tumor volume (length × width × height) were conducted every 2 days. After 3 weeks, mice were sacrificed for tumor analysis. All procedures were in compliance with our institution's guidelines for the use of laboratory animals and approved by the Penn State College of Medicine Institutional Animal Care and Use Committee.

### Statistical Analysis

Microarray statistical analysis was performed as describe [37]. Student t test was used comparing two groups. One-way ANOVA was used comparing multiple groups followed by Tukeys post-hoc test. All analysis with a p < 0.05 was considered significant.

## Results

### Mesenchymal cells acquire TISC characteristics post-EMT

**Figure 1:**
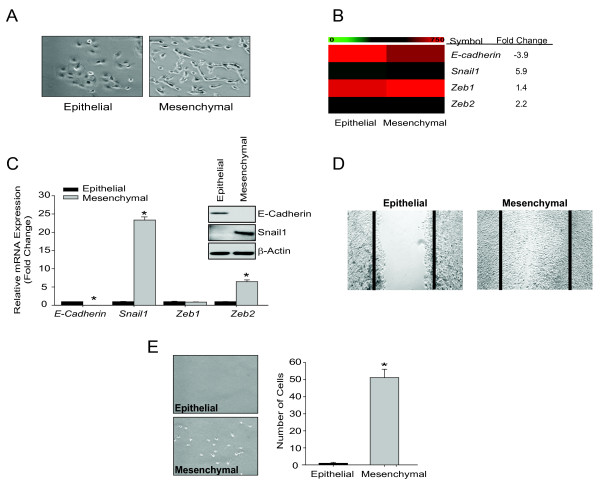
**Murine epithelial and mesenchymal liver cancer cells**. (A) Representative phase-contrast images (10 × magnification) of epithelial and mesenchymal cells. (B) Heatmap of mesenchymal markers generated from raw microarray values. (C) Relative expression of mRNAs endcoding *E-cadherin, Snail1, Ze*b1 and *Zeb2 *normalized to endogenous control *Gapdh*. Bars represent mean ± SEM of triplicates, *p < 0.01. Western blot analysis of E-cadherin, Snail1, and β-actin. Data representative of two independent experiments. (D) Wound healing assay. Bars represents mean ± SEM of triplicates, *p < 0.01. (E) Matrigel invasion assay. Phase-contrast images (4 × magnification) of cells that have invaded the membrane and adhered to the bottom of the plate. Data are expressed as the total number of invasive cells at the bottom of the plate, with five fields counted per well, n = 3 wells/cell line, and reported as the mean ± SEM, *p < 0.01.

In a previous report, we established a model of EMT using liver cancer cell lines derived from *Pten^-/- ^*mice [37]. In this model, we transplanted epithelial liver cancer cells, and from the resulting tumors, harvested epithelial and mesenchymal cells. The epithelial tumor cells were identical to parent cells, labeled P2-Epithelial (P2E), and the mesenchymal, fibroblastoid cells, were labeled P2-Mesenchymal (P2M) (Figure 1A). Both epithelial and mesenchymal cells demonstrated *Pten^-/- ^*genotype [37]. In support of the EMT-metastasis paradigm, mesenchymal cells demonstrated significant metastatic potential [37]. To confirm the persistence of epithelial and mesenchymal phenotypes, we analyzed the expression of key EMT genes and migratory/invasion *in vitro*. The mesenchymal cells demonstrate loss of E-cadherin, gain of E-box transcription repressors Snail1 and *Zeb2*, significant migration in wound assay, and increased invasion through Matrigel pores compared to epithelial cells (Figure 1B-E).

**Figure 2:**
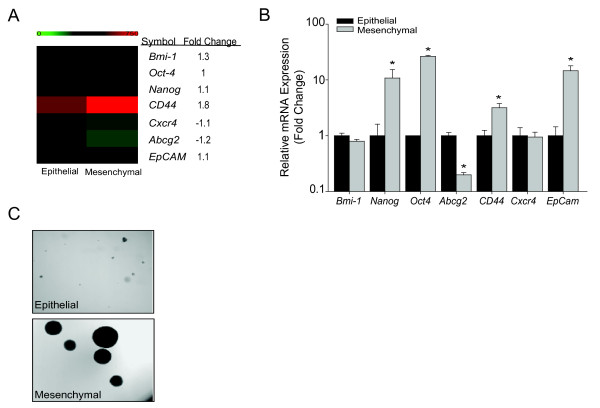
**Mesenchymal cells demonstrate up-regulation of TISC characteristics**. (A) Microarray heatmap of TISC markers. (B) Relative expression of mRNAs encoding TISC genes *Bmi-1, Nanog, Oct4, Abcg2, CD44, Cxcr4*, and *EpCam *normalized to endogenous control *Gapdh*. Data represent mean ± SEM of triplicates, *p < 0.01. (C) Tumor-sphere assay was performed for two weeks using non-adherent plates. Phase contrast images are representation of three independent experiments (4 × magnification).

In mesenchymal cells, transcriptome profiling demonstrated increased expression of multiple liver TISC markers (Figure 2A). Real-time PCR validated up-regulated *Nanog, Oct-4, CD44*, and *EpCam *(Figure 2B). Although CD133 is a strong TISC marker in previous reports, the mesenchymal cells have no detectable *CD133 *expression, making comparative analysis impossible. In terms of self-renewal assay, the mesenchymal cells were able to form large tumor-spheres in low adherent plates (Figure 2C). Increased stem cell markers and tumor-sphere formation indicates that the mesenchymal cells have a TISC phenotype.

### Resistance to chemotherapy is linked to cell proliferation

**Figure 3:**
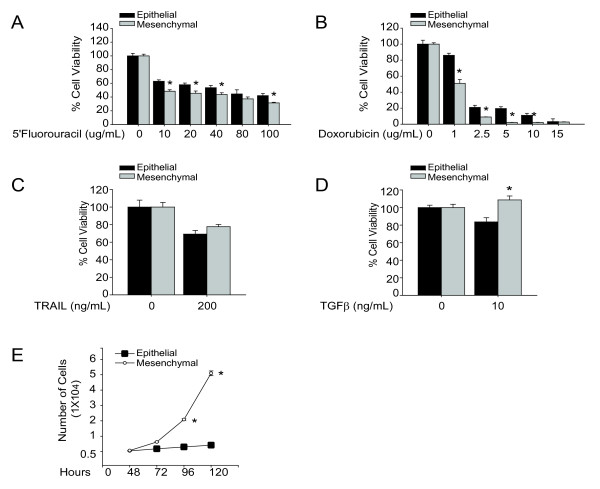
**Resistance to chemotherapy is linked to cell proliferation**. (A-D) Cell viability evaluation using XTT assay of cells treated with doxorubicin, 5'Fluorouracil, TRAIL or TGFβ for 48 hours. Data reported as mean ± SEM, n = 8, *p < 0.01. (E) Cell proliferation of epithelial and mesenchymal cells. 1 × 10^4 ^cells were plated on 60 mm^2 ^culture plates for 48 hours followed by cell count using cytometer at specific time points. Data are reported as mean ± SEM of triplicates, *p < 0.01.

To test the hypothesis that mesenchymal cells are resistant to chemotherapy, a TISC feature, cells were treated with doxorubicin and 5'Fluorouracil. The mesenchymal cells demonstrate increased sensitivity to genotoxic agents compared to epithelial cells (Figure 3A-B). In terms of cell cycle progression, the mesenchymal cells are highly proliferative compared to the epithelial cells (Figure 3E). Thus, we conclude that resistance to chemotherapy is linked to the level of cell proliferation, not mesenchymal status, consistent with the mechanism of action of cytotoxic agents. In addition to rate of proliferation, *Abcg2 *expression correlated with chemotherapy resistance (Figure 3A &3B, 2B), indicating that drug resistance may be dependent on the ATP-binding cassette expression as a mechanism of drug efflux. ATP-binding cassette efflux has been highly correlated to epithelial phenotype liver TISCs [14,42].

In addition to resistance to genotoxic agents, we assessed whether the mesenchymal cells are resistant to TRAIL-induced and TGFβ-induced apoptosis. Although there was no significant difference in response to TRAIL stimulation (Figure 3C), the mesenchymal cells demonstrate resistance to TGFβ-induced apoptosis (Figure 3D), a characteristic of TISCs [40].

### TGFβ-induced EMT results in TISC characteristics

**Figure 4:**
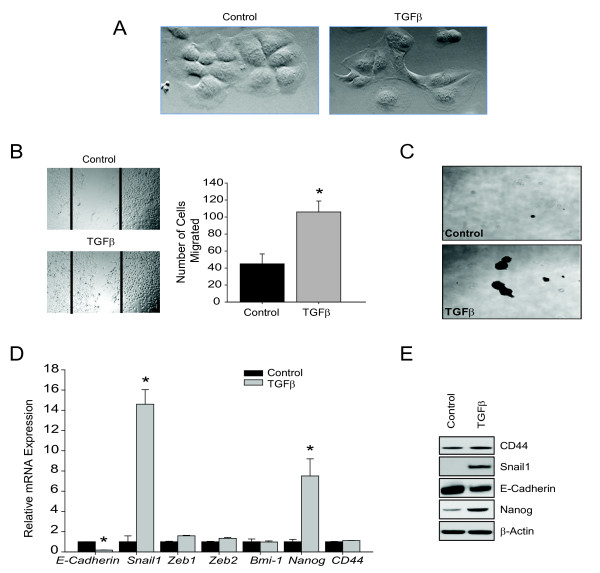
**TGFβ-induced EMT cells with CSC characteristics**. (A) Phase-contrast images of treated and untreated epithelial cells after 48 hours of TGFβ stimulation (20 ×). (B) Representative images of wound healing assay of TGFβ treated and untreated epithelial cells. Data represent mean ± SEM of triplicates, *p < 0.01. (C) Tumorsphere formation assay of TGFβ treated and untreated epithelial cells. Cells were cultured in low adherent plates for two weeks (4 × magnification). (D) Relative expression of mRNA encoding EMT and TISCs genes normalized to endogenous control *Gadph *after TGFβ stimulation. Data represent mean ± SEM of triplicates, *p < 0.01. (E) Western blot analysis of CD44, Snail1, E-Cadherin, Nanog, and *β-*actin.

During later stages of disease, TGFβ induces EMT and contributes to disease progression [15,43]. After TGFβ stimulation, epithelial cells undergo a morphological change from cuboidal to fibroblastic-like cells (Figure 4A). In addition to morphology change, TGFβ treatment resulted in increased cell migration and the formation of larger spheroids in low adherent plates (Figure 4B &4C. This TGFβ-induced change was associated with typical EMT characteristics, including decreased E-cadherin and increased Snail1 and Nanog (Figure 4D &4E).

### Inhibition of *Snail1 *blocks TISC characteristics

**Figure 5:**
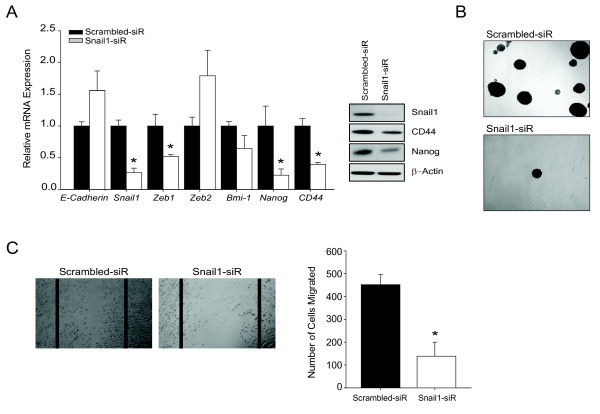
**Snail1 regulates EMT and CSC characteristics in mesenchymal cells**. (A) Epithelial cells were treated with *Snail1 *siRNA for 48 hours and mRNA expression was analyzed for *E-cadherin, Zeb1, Zeb2, Bmi-1, Nanog*, and *CD44 *normalized to *Gapdh*. Bars represent mean ± SEM of triplicates, *p < 0.01. Western blot analysis of Snail1, CD44, Nanog and β*-*actin, with data representative of two independent experiments. (B) Tumor-sphere formation assay of mesenchymal cells transfected with scrambled or *Snail1 *siRNA. Cells were cultured in low adherent plates (4 × magnification). (C) Wound assay of mesenchymal cells transfected with either scrambled or *Snail1 *siRNA. The number of cells migrated towards the wound was calculated. Data presented are mean ± SEM of triplicates, *p < 0.01.

In HCC, a TISC phenotype with Snail1 over-expression is associated with poor prognosis [21]. To test the specific role of Snail1 in up-regulating TISC characteristics, we utilized siRNA to knock down *Snail1 *in mesenchymal cells. After *Snail1 *siRNA treatment, TISC markers Nanog and CD44 decreased significantly (Figure 5A), which was associated with decreased spheroid formation (Figure 5B) and decreased migration (Figure 5C).

### TGFβ regulates Snail and Nanog through Smad signaling

**Figure 6:**
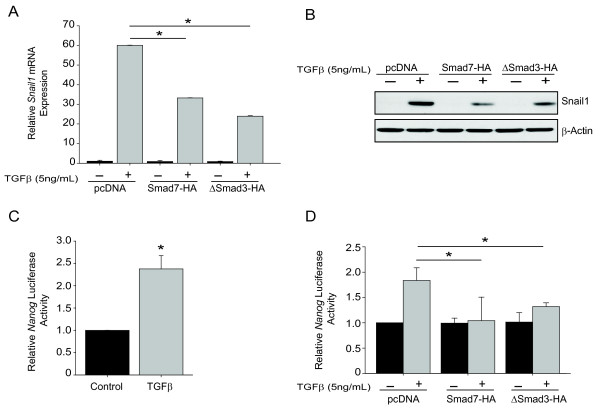
**TGFβ regulates Snail1 and Nanog through Smad signaling**. Epithelial cells were transfected with pcDNA control, Smad7 or ΔSmad3 and treated with TGFβ. (A) Relative *Snail1 *mRNA expression of epithelial cells. One-way ANOVA with Tukeys posthoc test was performed. Data shown as mean ± SEM of triplicates, *p < 0.01. (B) Western blot of Snail1 and β*-*actin. Data represent two independent experiments. (C) Epithelial cells were transfected with *Nanog-Luc *plasmid and treated with TGFβ. Luciferase activity was normalized to Renilla control. Data are shown as mean ± SEM of triplicates, *p < 0.05. (D) Relative *Nanog *luciferase activity after 24 hours of TGFβ stimulation. Data are shown as mean ± SEM of triplicates, *p < 0.05.

The primary mechanism of TGFβ-induced EMT is through Smad-dependent signaling. Following activation of TGFβ receptors, Smad2 and Smad3 are phosphorylated and form the Smad2/3/4 heterocomplex, which translocates to the nucleus to regulate *Snail1 *transcription [19,27,44]. After TGFβ stimulation in epithelial cells, Snail1 increased (Figure 4D). In order to confirm that TGFβ induces *Snail1 *through Smad-dependent pathways in our model, we utilized inhibitory Smads, Smad7 and dominant-negative Smad3 (ΔSmad3), which block heterocomplex formation. Epithelial cells were transfected with Smad7 or ΔSmad3 vectors 24 hours prior to TGFβ stimulation. qPCR and western blot analysis demonstrated that inhibitory Smads significantly attenuated TGFβ-induced Snail1 up-regulation (Figure 6A &6B).

TGFβ regulates *Nanog *promoter activity through Smad signaling in human embryonic stem cells [31]. To confirm that TGFβ can induce *Nanog *promoter activity in our model, epithelial cells were co-transfected with *Nanog-*Luc and Smad7 or ΔSmad3 vectors. Following TGFβ stimulation, *Nanog*-Luc activity was significantly attenuated by inhibitory Smads (Figure 6C &6D), indicating that TGFβ stimulates *Nanog *promoter activity through Smad-dependent signaling.

### Snail1 directly regulates Nanog promoter

**Figure 7:**
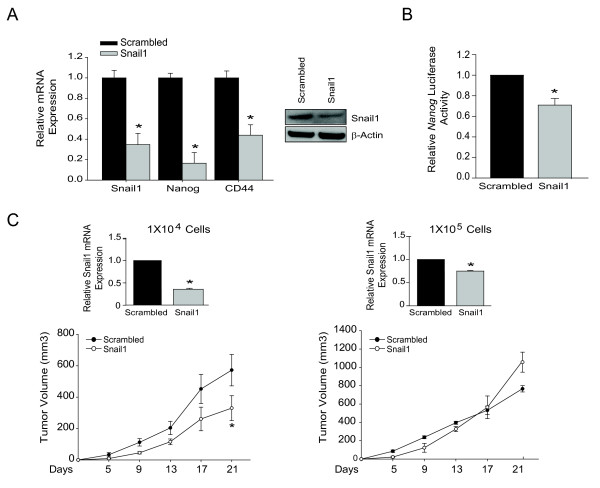
**Repression of Snail1 attenuates *Nanog *promoter activity and tumor proliferation**. (A) Relative gene expression of *Snail1, Nanog*, and *CD44 *of mesenchymal-scrambled-shRNA compared to mesenchymal-Snail1-shRNA cells. Bars represent mean ± SEM of triplicates, *p < 0.05. Western blot of Snail1 and β*-*actin, with blots representative of two independent experiments. (B) Inhibition of Snail1 reduces *Nanog *luciferase activity. Data presented are shown as mean ± SEM of three independent experiments, *p < 0.05. (C) Tumors of indicated number of cells of mesenchymal-scrambled-shRNA or mesenchymal-Snail1-shRNA knock-down cells. Tumor volume reported as mean ± SEM, *p < 0.05; N = 4/group. Relative *Snail1 *mRNA expression of tumor tissues. Data presented are reported as mean ± SEM of all Scrambled and Snail1 tumor tissues, *p < 0.05; N = 4/group.

After transient knock-down of *Snail1*, Nanog expression is decreased, indicating that Snail1 directly regulates TISC genes in mesenchymal cells (Figure 3B). To further investigate this Snail1-driven TISC expression profile, we established stable *Snail1 *knock-down in mesenchymal-Snail1-shRNA cells (Figure 7A). In these mesenchymal-Snail1-shRNA cells, down regulation of *Snail1 *corresponded to decreased *Nanog *promoter activity and decreased *Nanog *and *CD44 *expression (Figure 7A &7B).

### Inhibition of Snail1 results in decreased tumor growth *in vivo*

As demonstrated, Snail1 is a key regulator of TISC characteristics *in vitro*. To investigate the role of Snail1 in tumor initiation, we inoculated 1 × 10^4 ^mesenchymal-Snail1-shRNA cells into nude mice. The mesenchymal-Snail1-shRNA cells demonstrate reduced in tumor growth compared to control mesenchymal cells. Analysis of tumors demonstrates that *Snail1 *expression was down-regulated in 1 × 10^4 ^cell initiated tumors from mesenchymal-*Snail1*-siR cells (Figure 7C). However, tumor initiation was not affected by *Snail1 *suppression, as evidence by all inoculations forming tumors, even in *Snail1 *inhibited cells.

### Epithelial and mesenchymal differences in human HCC

**Figure 8:**
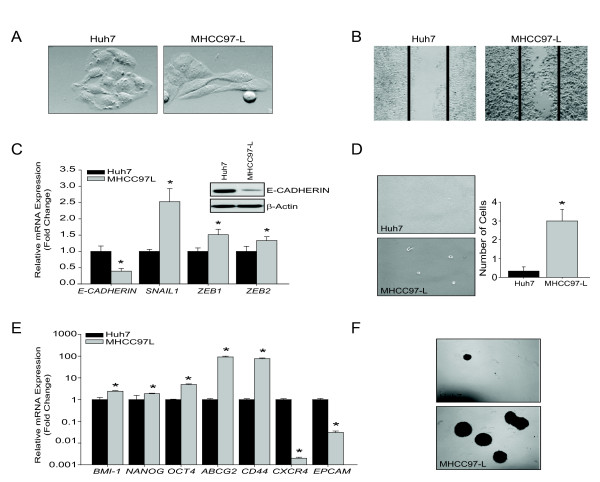
**Human epithelial and mesenchymal liver cancer cells**. (A) Representative phase-contrast images (20 × magnification) of Huh7 and MHCC97-L cells. (B) Representative images (4 × magnification) of Huh7 and MHCC97-L wound healing assay after 24 hours of scratching. Experiments were performed in triplicates. (C) Relative expression of mRNA endcoding *SNAIL1, ZEB1 *and *ZEB2 *normalized to endogenous control *GAPDH*. Bars represent mean ± SEM of triplicates, *p < 0.01. Western blot analysis of E-Cadherin and β-actin. Data representative of two independent experiments. (D) Matrigel invasion assay. Phase-contrast images (4 × magnification) of cells that have invaded the membrane and adhered to the bottom of the plate. Data are expressed as the total number of invasive cells at the bottom of the plate, with five fields counted per well, n = 3 wells/cell line, and reported as the mean ± SEM, *p < 0.01. (E) Relative expression of mRNA encoding TISC genes *BMI-1, NANOG, OCT4, ABCG2, CD44, CXCR4, and EPCAM *normalized to endogenous control *GAPDH*. Data represent mean ± SEM of triplicates, *p < 0.01. (F) Tumor-sphere assay was performed for two weeks using non-adherent plates. Phase contrast images are representation of three independent experiments (4 × magnification).

In order to investigate SNAIL1 and NANOG expression in human HCC cells, we utilized Huh7 and MHCC97-L cells. Huh7 cells have been described to be epithelial whereas MHCC97-L cells are mesenchymal with metastatic potential [38,39]. Accordingly, MHCC97-L cells demonstrate significant migration and invasion, increased expression of *SNAIL1, NANOG *and decreased expression of E-Cadherin (Figure 8B-D). Mesenchymal MHCC97-L cells also demonstrate TISC characteristics including increased *NANOG, BMI-1, CD44 and OCT4 *mRNA expression as well as increased tumorsphere formation (Figure 8E &8F).

## Discussion

Although liver transplantation has significantly improved survival in patients with early stage HCC, the prognosis for late stage HCC remains poor [45]. Causes of poor prognosis in late stage disease include invasive/metastatic disease and tumor recurrence after treatment. In breast cancer, EMT has been linked to TISC characteristics and resistant disease. Although this link between EMT and TISCs has been established in other cancers, including breast, prostate, nasopharyngeal, and colon cancer, this relationship has yet to be defined in HCC [17,22,46]. One potential link between EMT and TISCs in liver cancer is TGFβ.

TGFβ has a dual role in HCC either as a tumor suppressor in early stages or tumor promoter in later stages [15,43]. One of the mechanisms of early neoplastic transformation is through the evasion of cytostatic effects of TGFβ [43]. During the late stages of HCC tumorgenesis, TGFβ stimulates cellular invasion through the EMT program [44].

TGFβ induces EMT through *Snail1*, which represses *E-cadherin *by binding to E-box promoter elements [18,19,47]. In cancer patients, an EMT-phenotype transcriptome profile, with increased Snail1 expression, correlates with invasive tumors [21,48,49]. In this report, TGFβ stimulation of epithelial liver cancer cells results in a mesenchymal phenotype with fibroblastoid appearance, loss of *E-cadherin*, increased invasion and migration, and an up-regulation of Snail1. In addition, TGFβ treatment induces a TISC phenotype in epithelial cells. Although TGFβ-induced EMT generates TISC characteristics [17,22], the underlying mechanism has not yet been elucidated. Based on our results, we hypothesize that these TISC characteristics are Snail1 dependent. Inhibition of Snail1 causes the down-regulation of Nanog, *Bmi-1 *and CD44, loss of a migration and self-renewal as evidenced by decreased tumor-sphere formation.

Another key regulatory signaling pathway known to induce EMT in liver cells is the Hedgehog (Hh) signaling pathway. Hh promotes EMT in response to chronic liver injury [50]. In addition, Hh signaling has been suggested to play an important role in the maintenance of TISCs, and BMI-1, the polycomb group protein, may directly mediate Hh signaling in order to confer a self-renewal capacity in TISCs [10,46,51]. However, within our system, we were unable to see significant differences of BMI-1 between epithelial and mesenchymal cells.

TGFβ also directly controls Nanog in human embryonic stem cells [31]. Nanog is a key transcription factor that regulates self-renewal in stem cells [4,52]. Recent studies demonstrate that Nanog promotes TISC characteristics, and the down regulation of Nanog inhibits sphere formation and tumor development [4,34,35,53]. In this report, Nanog is up-regulated by TGFβ through Smad signaling. In addition, Snail1 directly regulates *Nanog *promoter activity.

TISCs are proposed to initiate tumors. In our model, liver cancer cells with a mesenchymal phenotype demonstrate TISCs characteristics, including tumor-sphere formation and increased expression of CD44 and Nanog. We further investigated epithelial and mesenchymal phenotypes in human HCC, Huh7 and MHCC97-L cells. Accordingly, Huh7 cells follow an epithelial phenotype whereas MHCC97-L cells are more mesenchymal demonstrating increased *Snail1, Zeb1, Zeb2 *mRNA expression, decreased E-cadherin expression, increased migration/invasion and increased tumorsphere formation [38].

In our murine system, *Snail1 *inhibition resulted in loss of tumor-sphere formation, decreased expression of CD44 and Nanog, and decreased tumor growth. According to our *in vitro *results, Snail1 clearly regulates TISC characteristics. However, the loss of Snail1 is not sufficient to inhibit tumor initiation, as evidenced by *in vivo *results. These findings are not un-expected in that the proposed TISC-driven tumor initiation is an early event in tumorigenesis, and cells that acquire TISC characteristics after EMT are a late event in tumor progression. In addition, Snail1 is one of many regulators of EMT, and thus manipulation of multiple factors may be required to fully inhibit tumor initiation.

## Conclusion

In summary, we demonstrated that TGFβ induces EMT and TISC characteristics through the up-regulation of Snail1 and Nanog. In addition, Snail1 directly regulates *Nanog *promoter activity. Notably, expression of both SNAIL1 and NANOG is higher in human mesenchymal cells. Inhibition of Snail1 alone is not sufficient to inhibit tumor initiation, but does result in reduction of tumor growth *in vivo*.

## List of abbreviations

TISCs: Tumor initiating stem-like cells; HCC: hepatocellular carcinoma; EMT: epithelial-mesenchymal-transition; TGFβ: transforming growth factor-β; FBS: fetal bovine serum.

## Competing interests

Dr. Rountree reports research support of less than $10,000 from Bayer Pharmaceuticals for un-related studies. Authors Dang, Ding, and Emerson report no competing interests.

## Authors' contributions

HD carried out the molecular and in vivo studies and drafted the manuscript. WD assisted in molecular and in vivo studies and manuscript preparation. DE participated in molecular in vitro studies. CBR conceived of the study, and participated in its design and coordination and helped to draft the manuscript. All authors read and approved the final manuscript.

## Pre-publication history

The pre-publication history for this paper can be accessed here:

http://www.biomedcentral.com/1471-2407/11/396/prepub
